# The Neuromeric/Prosomeric Model in Teleost Fish Neurobiology

**DOI:** 10.1159/000525607

**Published:** 2022-06-21

**Authors:** Mario F. Wullimann

**Affiliations:** ^a^Division of Neurobiology, Department Biologie II, Ludwig-Maximilians-Universität München (LMU Munich), Martinsried, Germany; ^b^Department Genes-Circuits-Behavior, Max-Planck-Institute for Biological Intelligence (i.F.), Martinsried, Germany

**Keywords:** Dopamine system, Eminentia thalami, Medial amygdala, Preglomerular complex, Preoptic region, Pretectum, Radial migration, Subpallium, Tangential migration, Thalamus, Zebrafish

## Abstract

The neuromeric/prosomeric model has been rejuvenated by Puelles and Rubenstein [Trends Neurosci. 1993;16(11):472–9]. Here, its application to the (teleostean) fish brain is detailed, beginning with a historical account. The second part addresses three main issues with particular interest for fish neuroanatomy and looks at the impact of the neuromeric model on their understanding. The first one is the occurrence of four early migrating forebrain areas (M1 through M4) in teleosts and their comparative interpretation. The second issue addresses the complex development and neuroanatomy of the teleostean alar and basal hypothalamus. The third topic is the vertebrate dopaminergic system, with the focus on some teleostean peculiarities. Most of the information will be coming from zebrafish studies, although the general ductus is a comparative one. Throughout the manuscript, comparative developmental and organizational aspects of the teleostean amygdala are discussed. One particular focus is cellular migration streams into the medial amygdala.

Prologue:

*Defendi rem publicam adulescens,*

*non deseram senex*

(Cicero, Phil II, 118)

This is a very personal account on how the author got involved with zebrafish brain research and contributed to introduce the neuromeric/prosomeric model into this field.

## Part 1: The Basics

This first of two parts introduces the main characteristics of the amniote neuromeric model and leads over to its application in teleost fish brains.

### Pillars of the Neuromeric/Prosomeric Model

Although the discussion of body and brain segmentation is much older, it was the seminal paper by Puelles and Rubenstein [1993] that rejuvenated the topic of brain transverse units (segments, neuromeres). These authors summarized how various socioscientific conditions had led to wide-held views that ignored or dismissed information on vertebrate brain segmentation. Their newly proposed neuromeric/prosomeric model [Puelles and Rubenstein, 1993] was initially established for the amniote brain and integrates the interdigitating topology of classical central nervous system (CNS) longitudinal domains (that is, floor, alar, basal, and floor plates) with transverse elements (neuromeres, segments) along the anteroposterior neural tube axis. A main stake of a neuromere is that it includes all four dorsoventral longitudinal zones just mentioned. The neuromeric model quickly proved highly advantageous for topological and developmental vertebrate brain analyses − in particular for cross-species comparisons − and, in its wake, influenced hundreds of publications.

**Fig. 1:**
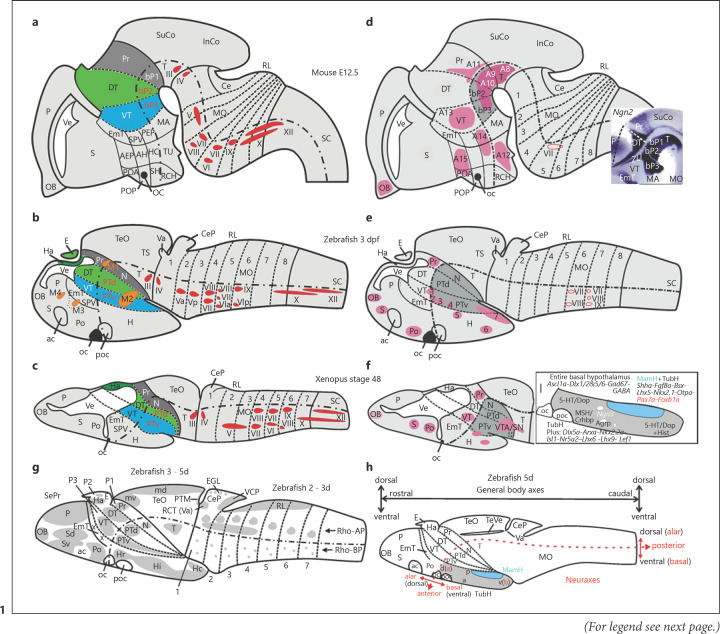
Brain schematics in lateral view for amniotes (**a, d**), teleosts (**b, e**) and amphibians (**c, f**) point out neuromeric divisions. Left side panels [**a–c**; adapted from Wullimann, 2020] emphasize forebrain with pretectal (P1) prosomere in dark gray, (dorsal) thalamic (P2) prosomere in green and ventral thalamic/prethalamic (P3) in blue. Note that for reasons given in the text, an early version of the amniote prosomeric model [Puelles and Rubenstein, 1993] is given in (**a, d**). In contrast, the zebrafish model follows in general that proposed by Wullimann and Puelles [1999]. Positions of early migrated teleostean forebrain areas M1 through M4 are highlighted (orange structures in B). In midbrain and hindbrain (**a–c**), primary neuromeric locations of motor nuclei (red) are shown [after Gilland and Baker, 2005, zebrafish efferent octavolateralis and facial motor neurons after Beiriger et al., 2021]. Right side panels [**d–f**; adapted from Vernier and Wullimann, 2009] emphasize basal plate portions of prosomeres (bPs in dark gray) and dopamine systems in midbrain and forebrain (pink structures) interpreted within the neuromeric model. Mouse A8-A15 dopamine cell groups correspond to the nomenclature of Smeets and González [2000] and Björklund and Dunnett [2007]. The Arabic numbers of zebrafish dopamine cell groups are taken from Rink and Wullimann [2002]. *Xenopus* dopamine nuclei are according to González et al. [1994a, b], González and Smeets [1994], Smeets and González [2000], and Xavier et al. [2017]. The *inset* in (**d**) shows *Ngn2* expression in the mouse diencephalon at this sagittal section level (modified from Osório et al. [2010]; see text). In the hindbrain (**d, e**) secondary (tangentially migrated) positions of various motor nuclei (red-rimmed) are shown [after Gilland and Baker, 2005; zebrafish efferent octavolateralis and facial motor neurons after Beiriger et al., 2021]. Prosomeric and rhombomeric boundaries are indicated by dashed lines. **g** Postembryonic zebrafish brain proliferation zones visualized either with PCNA [Wullimann and Puelles, 1999; Wullimann and Knipp, 2000] or BrdU [Mueller and Wullimann, 2002a] support prosomeric model [Puelles and Rubenstein, 1993]. Modified from Mueller and Wullimann [2016]. Alar plate (dorsal) and basal plate (ventral) are separated by a chain line along the anteroposterior axes (note flexures deviating from general body axis; see text). **h** Schema shows larval brain zebrafish brain schema in lateral view with indication of general body axes on top and in red the anteroposterior and dorsoventral (alar-basal) neuraxes that respect the brain curvature [modified from Herget et al., 2014]. Additionally, intrahypothalamic neuraxes are indicated, and tuberal (gray) and mammillary (light blue) basal hypothalamic parts are highlighted (see text for details). **i** Larval zebra­fish basal hypothalamus with various landmark-providing markers [after Wang et al. [2001]; Rink and Guo [2004]; Forlano and Cone [2007], general basal hypothalamic gene expression (see text for citations) and specific tuberal (TubH) and mammillary hypothalamic (MamH) gene expression after Schredelseker and Driever [2020]. a, anterior; ac, anterior commissure; AEP, anterior entopeduncular area (mouse); Agrp, Agouti-related protein; AH, anterior hypothalamus (mouse); bP1-3, basal parts of prosomeres 1–3; Ce, cerebellum; CeP, cerebellar plate; Crhbp, corticotropin-releasing hormone binding protein; d(a), dorsal(alar); Dop, dopamine; DT, dorsal thalamus; E, epiphysis; EGL, external granular layer; EmT, eminentia thalami; H, hypothalamus; Ha, habenula; Hc, Hi, Hr, caudal, intermediate, rostral periventricular hypothalamic zone; HC, hypothalamic cell cord (mouse); Hist, histamine; InCo, inferior colliculus; M1, early migrated pretectal aera; M2, early migrated posterior tubercular area (preglomerular complex); M3, early migrated area of eminentia thalami; M4, early migrated telencephalic area; MA, mammillary hypothalamus (mouse); MamH, mammillary hypothalamus (zebrafish); md, mediodorsal tectal proliferation; MO, medulla oblongata; MSH, α-melanocyte-stimulating hormone; mv, medioventral tectal proliferation; N, area of the nucleus of the medial longitudinal fascicle; OB, olfactory bulb; oc, optic chiasm; p, posterior; P, pallium; P1-P3, prosomeres 1–3; PEP, posterior entopeduncular area (mouse); Po, preoptic region; POA, anterior preoptic area (mouse); poc, postoptic commissure; POP, posterior preoptic area (mouse); Pr, pretectum; PTd, dorsal posterior tuberculum; PTv, ventral posterior tuberculum; PTM, posterior tectal membrane; RCH, retrochiasmatic hypothalamus (mouse); RCT, rostral cerebellar thickening (valvula); Rho-AP, alar plate proliferation of rhombencephalon; Rho-BP, basal plate proliferation of rhombencephalon; RL, rhombic lip; S, subpallium; SC, spinal cord; Sd, dorsal division of subpallium; SePr, secondary prosencephalon; SH, suprachiasmatic area (mouse); SPV, supraopto-paraventricular area; SuCo, superior colliculus; Sv, ventral division of subpallium; T, midbrain tegmentum; TeO, tectum opticum; TeVe, tectal ventricle; TS, torus semicircularis; TU, tuberal hypothalamus (mouse); TubH, tuberal hypothalamus (zebrafish); v(b), ventral(basal); Va, valvula cerebelli; VCP, ventral cerebellar proliferative layer; Ve, forebrain ventricle; VT, ventral thalamus (prethalamus); VTA/SN, ventral tegmental area/substantia nigra, x location of ventricular proliferation zone of EmT; ZLI, zona limitans intrathalamica. 1–8 rhombomeres 1 through 8, additionally in panel e: 1–7 designate larval zebrafish dopaminergic cells groups (see text for details), III, IV,V (Va/Vp), VI (VIa/VIp), VII, VIII, IX, X, XII ocolumotor, trochlear, trigeminal (anterior/posterior trigeminal), abducens (anterior/posterior abducens), facial, octavolateralis efferent, glossopharyngeal, vagal, hypoglossal motor nuclei, 5-HT 5-hydroxytryptamine.

One main reason for the neuromeric model's success is that it is based on early **developmental gene expression patterns** and their roles for brain development [Puelles and Rubenstein, 1993]. Various locally emitted morphogenes near (e.g., from skin, somites, notochord) and within the neuroepithelium (e.g., from midbrain-hindbrain boundary, roof plate, floor plate) before and during the transitory embryonic neuromeric phase initiate dynamic differential gene expression in the vertebrate neural tube. This first leads to cross- and autoregulatory as well as repressive gene activity in the neural tube which results, for example, in the formation of a series of hindbrain neuromeres (i.e., rhombomeres 1 through 8 in Fig. 1) having interrhombomeric boundaries (reviews [Cordes, 2001; Alexander et al., 2009]). Finally, various *Hox* cluster genes are expressed in an anteroposterior hierarchical fashion in rhombomeres 1 through 8 or genes are specifically expressed in particular rhombomeres (e.g., *Krox20* in 3/5, *Hoxb1* in 4), always respecting neuromeric boundaries. Subsequently, this differential gene expression leads to the identity and fate of cells constituting each rhombomere [Holland and Hogan, 1988; Wilkinson and Krumlauf, 1990; Hunt et al., 1991; Hunt and Krumlauf, 1992; Krumlauf et al., 1993; Lumsden and Krumlauf, 1996].

A major issue for the neuromeric model is **segmental boundaries.** In addition to gene expression, a second major argument for recognizing vertebrate brain neuromeres is clonal cell lineage restriction, which is itself the result of differential rhombomeric gene expression noted above. There is a considerable body of work showing experimentally during this early embryonic neuromeric time period, when amniote rhombomeres (1–8 in Fig. 1) are factually seen even morphologically, that cell clones of one rhombomere in general do not cross interrhombomeric boundaries [Fraser et al., 1990; Larsen et al., 2001], except for a small fraction of cells [Birgbauer and Fraser, 1994]. This is of critical importance during the initial development of motor nuclei in the rhombencephalon (Fig. 1a-c motor nuclei after [Gilland and Baker, 2005]; zebrafish efferent octavolateralis and facial motor neurons after [Beiriger et al., 2021]). However, this process can be experimentally manipulated by altering gene expression, which leads to predictably different motor neuron identities [Lumsden, 2004; Kiecker and Lumsden, 2005], directly demonstrating the role of neuromere-specific gene activity for cell fate.

Thus, the primary rhombomeric origin of some cranial nerve motor nuclei is identical interspecifically (e.g., IV in 1, V in 2/3, VI in 5, VII in 4, IX in 6; see Fig. 1a-c). However, zebrafish and frog (*Xenopus*) facial and zebra­fish abducens motor neurons originate additionally in the respective posteriorly adjacent rhombomere. What is more, motor neurons may later migrate in the postneuromeric stage tangentially into more posterior rhombomeric locations (Fig. 1d, e; migrated motor nuclei after [Gilland and Baker, 2005]; zebrafish efferent octavolateralis and facial motor neurons after [Beiriger et al., 2021]), for example, mouse facial motor neurons into 5/6 (Fig. 1d) or zebrafish facial/octavolateralis and glossopharyngeal motor neurons into 6/7 and 7, respectively (Fig. 1e). Without the prosomeric/neuromeric concept, these interspecific differences in motor neuron primary origin and subsequent dynamic developmental changes in location would almost be impossible to understand [Kinkhabwala et al., 2011].

The neuromeric/prosomeric model also recognizes transverse elements in the amniote forebrain (prosomeres, initially six in number [Puelles and Rubenstein, 1993]), but they have remained somewhat more controversial than rhombomeres (review in [Wullimann, 2017]). There is evidence for a forebrain-midbrain boundary [Larsen et al., 2001] and the zona limitans intrathalamica (inset in Fig. 1d) forms a transverse boundary between dorsal and ventral thalamus [Zeltser et al., 2001]. While clonal cell lineage restriction of rhombomeres has been well established (see above), there is much less comparable information in the amniote forebrain. However, similar clonal cell labeling experiments in the chick showed that a ventral thalamic, dorsal thalamic, and pretectal neuromere/prosomere (the latter bisected by the posterior commissure) do exist [Figdor and Stern, 1993]. Fate map studies using quail-chick grafts also show the early existence of these three diencephalic prosomeres [García-López et al., 2009]. Furthermore, various genes are expressed in specific amniote prosomeres, *Prox* in pretectum, *Gbx2* in dorsal thalamus, *Dlx2* in ventral thalamus ([Larsen et al., 2001]; see also zebrafish data in: The specifics). All this supports the recognition of three amniote prosomeres in the diencephalon, i.e., from posterior to anterior, a pretectal (P1), dorsal thalamic (thalamic) (P2) and ventral thalamic (prethalamic) (P3) one, classically referred to as pretectum, dorsal thalamus, and ventral thalamus. Anterior to P3 (prethalamus) is a large and complexly organized transverse unit that includes the entire hypothalamus and telencephalon, termed the secondary prosencephalon (for more information, see: The specifics).

An equally important aspect of the neuromeric/prosomeric model is the direct demonstration of the true **longitudinal (anteroposterior) axis** of the CNS through longitudinal gene expression patterns, such as that of the ventrally expressed signaling factor coding gene *Sonic hedgehog* (*Shh*) [Shimamura et al., 1995]. Thus, two main deflections from the general anteroposterior body axis occur in the amniote brain: one in a dorsal direction in the anterior hindbrain and one in the opposite direction in the forebrain (see course of chain line in mouse brain example in Fig. 1a, d) with the ventrally directed forebrain deflection being obvious in anamniote brains as well (see Fig. 1b, c, e, f). This is a major conceptual change deviating from previous views (columnar models; see discussion in [Puelles and Rubenstein, 1993]). Together with the transverse gene expression patterns mentioned above, the longitudinal ones allow for adequate topological allocation of neural structures. For example, the early basal (ventral) forebrain is directly opposite to the basal hindbrain. This is paramount for understanding dorsoventral and ventrodorsal signaling and its resulting major differentiation effects along the entire neural tube. The model also allows for understanding the true topological relationship of the diencephalon, whose major parts form an anteroposterior series and not a ventrodorsal one, as interpreted in much of classical neuroanatomy. Thus, the hypothalamus (H) is anterior (not ventral) to the ventral thalamic prosomere, which is thus newly interpreted as prethalamus because it lies anterior to the dorsal thalamic prosomere (thalamus proper). The latter, in turn, lies anterior to pretectum (Pr; Fig. 1a). Since the hypothalamus (alar and basal plate portions; Fig. 1a) belongs together with the telencephalon to the same large, most anterior CNS division (i.e., the secondary prosencephalon), the traditional term “diencephalon” designates therefore not a true transverse unit and is newly used for P1 through P3 only. However, for didactic reasons, the author continues here to use the terms dorsal and ventral thalamus (DT/VT) in the traditional sense, i.e., designating the alar parts of these two prosomeres, respectively, and excluding the habenula and pineal/parietal from this usage of DT.

### The Neuromeric Model in Fish Brain Research

Initiated by the Oregon School of George Streisinger and collaborators during the 1970s and 1980s (e.g. [Strei­singer et al., 1981]), the zebrafish *Danio rerio* (formerly *Brachydanio rerio*) has become the main developmentally studied fish species. Thus, much of the following will be dealing with this animal, but always with a focus on comparative vertebrate brain aspects. However, the author will not address cartilaginous fish and agnathans. Our own work involved the analysis of the entire zebra­fish brain and sometimes also focused on hindbrain issues, such as cerebellar, rhombic lip and raphe development ([Lillesaar et al., 2009; Volkmann et al., 2010]; review [Wullimann et al., 2011; Biechl et al., 2016]). However, the author will restrict himself below to comparative forebrain issues. For details on the olfactory bulb and dorsal telencephalic area (i.e., pallium), the author refers to our various other publications [Wullimann and Mueller, 2004a; Mueller and Wullimann, 2009; Wullimann, 2009; Wullimann and Vernier, 2009a; b; Mueller and Wullimann, 2016; Wullimann, 2017; Gerlach and Wullimann, 2021]. Furthermore, there is a recent ambitious examination of pallial (and subpallial) divisions in the zebrafish [Porter and Mueller, 2020]; some of its more speculative arguments will be considered below (The specifics).

Some of the mentioned Oregon school's earliest zebrafish neurobiological work focused on the development of serially homologous midbrain and hindbrain structures (e.g., reticulospinal and other segmentally repeated neurons [Kimmel et al., 1982; 1985; 1995; Mendelson 1986a; b; Metcalfe et al., 1986, 1990; Hanneman et al., 1988; Trevarrow et al., 1990; Kimmel, 1993]). Thus, the segmental (neuromeric, rhombomeric) nature of the hindbrain gained early recognition in the field. Also, much attention was paid by the community on early embryonic (i.e., pre-neuromeric) zebrafish CNS development, such as pioneer neurons and the early axonal scaffold (e.g. [Chitnis and Kuwada, 1990; Wilson et al. 1990; Ross et al., 1992]). After molecular genetics revolutionized the zebrafish field in the early 1990ties (reviews by [Mullins and Nüsslein-Volhard, 1993; Mullins et al., 1994; Postlethwait et al., 1994; Solnica-Krezel et al., 1994]), gene expession and functional genetic studies accelerated and major progress was made regarding the genoarchitectonics of zebrafish rhombomeric development (e.g., [Niølstad and Fjose, 1988; Oxtoby and Jowett, 1993; Moens et al., 1998; Prince, 1998; Prince et al., 1998; Kimmel et al., 2001]). However, prosomeres were not addressed in this early zebrafish work.

**Fig. 2:**
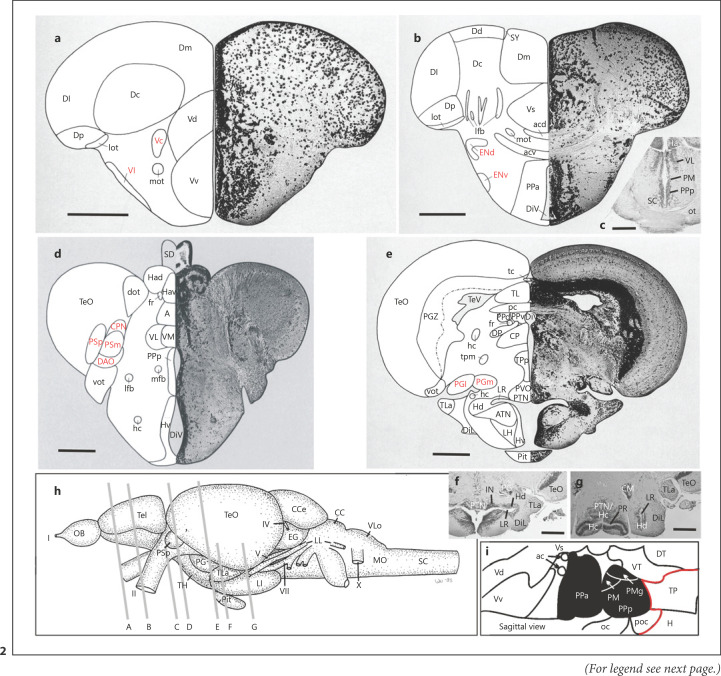
Zebrafish adult forebrain neuroanatomy shown in transverse Bodian silver/cresyl stained sections [panels modified from Wullimann et al., 1996, see there for full account]. **a** Precommissural telencephalon. **b** Commissural telencephalon level. **d** Rostral diencephalon. **e** Caudal diencephalon. These four levels show peripherally migrated forebrain cell areas (highlighted with red letters), such as subpallial (Vc, Vl), entopeduncular (ENd, ENv), pretectal (CPN, PSp, PSm, DAO) and preglomerular nuclei (PGl, PGm) (see text). **c** Shows preoptic region in between (**b, d**). **f** Slightly more caudal level than (**e**) with intermediate nucleus of Hd (see text). **g** Most caudal diencephalic section with posterior and lateral recess at the same transverse level of caudal hypothalamus (Hc; see text). **h** Lateral view of adult zebrafish brain with transverse section levels indicated. **i** Shows drawing of parasagittal section through zebrafish preoptic region (black) [modified from Herget et al., 2014]. Note that the suprachiasmatic nucleus (shown in **c**) is lateral to the section level. Red line is alar-basal plate boundary. Scale bars, 200 µm. A, anterior thalamic nucleus; ac, anterior commissure; acd, acv, dorsal, ventral anterior commissure; APN, accessory pretectal nucleus [of Wullimann and Meyer [1990]; ATN, anterior tuberal nucleus; CC, cerebellar crest; CCe, corpus cerebelli; CM, corpus mamillare; CP, central posterior thalamic nucleus; CPN, central pretectal nucleus; DAO, dorsal accessory optic nucleus; Dc Dd, Dl, Dm, Dp, central, dorsal, lateral, medial, posterior zone of dorsal telencephalic area; DiL, diffuse nucleus of the inferior lobe; DiV, diencephalic ventricle; dot, dorsomedial optic tract; DP, dorsal posterior thalamic nucleus; DT, dorsal thalamus; EG, eminentia granularis; ENd, ENv, dorsal, ventral entopeduncular nucleus; fr, fasciculus retroflexus; H, (basal) hypothalamus; Ha, habenula; Had, dorsal habenular nucleus; Hav, ventral habenular nucleus; hc, horizontal commissure; Hc, Hd, Hv, caudal, dorsal, ventral zone of periventricular hypothalamus; IN, intermediate nucleus of Hd; lfb, lateral forebrain bundle; LH, lateral hypothalamic nucleus; LI, lobus inferior; LL, lateral line nerves; lot, lateral olfactory tract; LR, lateral recess of diencephalic ventricle; mfb, medial forebrain bundle; MO, medulla oblongata; mot, medial olfactory tract; OB, olfactory bulb; oc, optic chiasma; ot, optic tract; pc, posterior commissure; PG, preglomerular complex; PGI, lateral preglomerular nucleus; PGm, medial preglomerular nucleus; Pit, pituitary; PM, magnocellular preoptic nucleus; PMg, gigantocellular part of PM; poc, postoptic commissure; PPa, anterior parvocellular preoptic nucleus; PPd, dorsal periventricular pretectal nucleus; PPp, posterior parvocellular preoptic nucleus; PPv, ventral periventricular pretectal nucleus; PSm, magnocellular superficial pretectal nucleus; PSp, parvocellular superficial pretectal nucleus; PTN, posterior tuberal nucleus; PVO, paraventricular organ; SC, suprachiasmatic nucleus (spinal cord in H); SD, saccus dorsalis; SY, sulcus ypsiloniformis; tc, tectal commissure; Tel, telencephalon; TeO, tectum opticum; TeV, tectal ventricle; TH, tuberal hypothalamus; TL, torus longitudinalis; TLa, torus lateralis; TP, posterior tuberculum; TPp, periventricular nucleus of TP; tpm, tractus pretectomamillaris; Vc, Vd, Vl, Vs, Vv, central, dorsal, lateral, supracommissural, ventral nucleus of ventral telencephalic area; VL, ventrolateral thalamic nucleus; VLo, vagal lobe; VM, ventromedial thalamic nucleus; vot, ventrolateral optic tract; VT, ventral thalamus (prethalamus); I, olfactory nerve; II, optic nerve; IV, trochlear nerve; V, trigeminal nerve; VII, facial nerve; X, vagal nerve.

Harry Bergquist [Bergquist, 1932, 1954; Bergquist and Källén, 1954], a member of the Swedish Comparative Embryological School, early proposed forebrain neuromeres in addition to hindbrain neuromeres and included various fish species for documentation (see reviews [Puelles and Rubenstein, 1993; Vernier and Wullimann, 2009; Mueller and Wullimann, 2016]). To my knowledge, our adult brain atlas “Neuroanatomy of the Zebrafish Brain” ([Wullimann et al., 1996]; Figure 2) was the first publication that explicitely discussed the prosomeric model of Puelles and Rubenstein [1993] in a zebrafish context. My subsequent personal entry point into developmental zebrafish brain research was the realization that proliferation patterns are at the core of the consideration of neuromeres and form the basis for understanding brain genoarchitectonics discussed in the second part (The specifics). Proliferation zones are also paramount to understand histogenetic units, i.e., [Puelles and Medina, 2002] defined as all cellular derivatives originating from a particular proliferation zone. Therefore, we next undertook a thorough analysis of early (2–5 days) zebrafish brain proliferation patterns (Fig. 1g), first visualizing the proliferating cell nuclear antigen (PCNA) [Wullimann and Puelles, 1999; Wullimann and Knipp, 2000], and later confirming the data using a BrdU strategy [Mueller and Wullimann, 2002a]. The working hypothesis was that during the neuromeric period the pattern of proliferation zones should show neuromeric brain organization. Indeed, in addition to neuromeric patterns of alar and basal plate proliferation zones in rhombomeres, discreet proliferation zones in both alar and basal pretectum (P1), dorsal thalamus (P2), and ventral thalamus (P3) were obvious (Fig. 1g). This suggested a three-prosomere model (Fig. 1) and was then first proposed for the zebra­fish brain based on these early proliferation patterns [Wullimann and Puelles, 1999]. The larval zebrafish brain proliferation patterns anterior to ventral thalamus are highly complex and not easily interpreted in a prosomeric fashion. Thus, the initially proposed three anterior-most prosomeres (including telencephalon and hypothalamus) were newly considered to represent a large and complex so-called secondary prosencephalon (SePr in Fig. 1g) with many subdivisions that are not obviously prosomeric in nature [Wullimann and Puelles, 1999]. We also proposed a similar three-prosomere model for *Xenopus* (Fig. 1c; after [Wullimann et al., 2005]). Moreover, together with follow-up studies involving genes active in neurogenesis, we concluded that a new (secondary) wave of neurogenesis − after a primary embryonic one − emerges during the neuromeric period (discussed in [Mueller and Wullimann, 2016]).

**Fig. 3:**
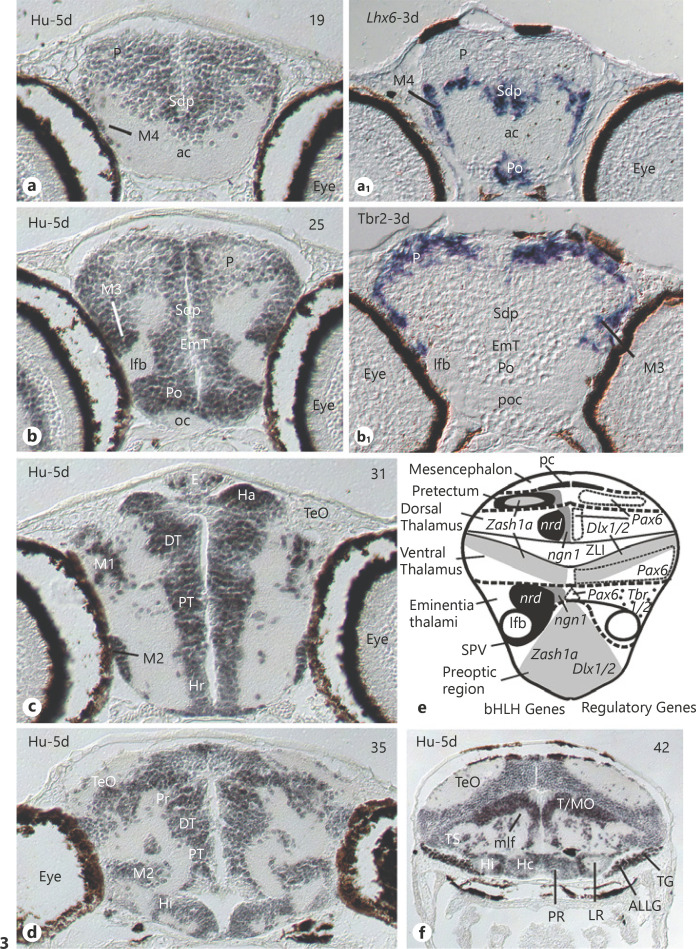
**a–d** Zebrafish larval forebrain neuroanatomy shown in transverse Hu-protein stained sections. **a** Commissural telencephalon with early migrated telencephalic area M4. **b** Postcommissural telencephalon with early migrated area of eminentia thalami M3. **c** Rostral diencephalon with early migrated posterior tubercular area M2 (preglomerular complex) and early migrated pretectal area M1. **d** Caudal diencephalon with early migrated posterior tubercular area M2 (preglomerular complex). **e** Summary of larval diencephalic zebrafish gene expression patterns (see text for details). **f** Posteroventral hypothalamic level shows lateral and posterior ventricular recess. Panels (**a–f**) modified from Mueller and Wullimann [2016]; see there for full account. **a1–b1** Corresponding sections with diagnostic regulatory gene markers to identify larval zebrafish migrated areas M4 and M3 [panels modified from Mueller et al., 2008]. ac, anterior commissure; ALLG, anterior lateral line ganglion; bHLH, basic helix-loop-helix; DT, dorsal thalamus; E, epiphysis; EmT, eminentia thalami; Ha, habenula; Hc, Hi, Hr, caudal, intermediate, rostral periventricular hypothalamic zone; lfb, lateral forebrain bundle; LR, lateral ventricular recess of periventricular hypothalamus; M1, early migrated pretectal aera; M2, early migrated posterior tubercular area (preglomerular complex); M3, early migrated area of eminentia thalami; M4, early migrated telencephalic area; mlf, medial longitudinal fascicle; MO, medulla oblongata; oc, optic chiasma; P, pallium; pc, posterior commissure; Po, preoptic area; poc, postoptic commissure; Pr, pretectum; PR, posterior ventricular recess of periventricular hypothalamus; PT, posterior tuberculum; Sdp, posterior subdivision of dorsal part of subpallium (subpallial amygdala homolog); SPV, supraopto-paraventricular region; T, midbrain tegmentum; TeO, tectum opticum; TG, trigeminal ganglion; TS, torus semicircularis; ZLI, zona limitans intrathalamica. For gene names see text.

Thus, we fastly arrived at a first genoarchitectonic segmentation of the diencephalon (summarized in [Wullimann and Mueller, 2004a]; Fig. 3e) and much of it will be discussed below (see: The specifics). A main result of our initial gene expression analyses was that most brain areas are either characterized by gene expression leading to GABAergic cells (e.g., subpallium, alar ventral alamus, hindbrain GABA positive stripes) or by different gene expression leading to the glutamatergic cell phenotype (pallium, alar dorsal thalamus). Other areas (e.g., alar pretectum, basal plate diencephalon) express markers of both categories but always in different cells (see more details below).

Of note, the eminentia thalami (EmT) represents prosomere 4 in the initial amniote prosomeric model [Puelles and Rubenstein, 1993]. In the zebrafish, we saw a small proliferation zone sandwiched between the large ventral thalamic and the preoptic proliferation zones and suggested that it might be the proliferative zone of the EmT [Wullimann and Puelles, 1999]. This was later confirmed in detail [Wullimann and Mueller, 2004b; Mueller et al., 2008] with very specific gene expression (see: The specifics). Thus, the author considers the EmT part of the secondary prosencephalon and neither a prosomere in its own right (as in [Puelles and Rubenstein, 1993]) nor part of P3 (as in [Puelles and Rubenstein, 2003]) and the schematics used (Fig. 1) follow this view. The terminology shown in the schema for the mouse (Fig. 1a, g) stem from the original paper by Puelles and Rubenstein [1993] because this explanatory schema is used in several of our and other publications discussed here. Thus, the author keeps this schema here in order to relate intelligibly and lucidly to this previous literature.

As discussed above for amniotes, there are various requirements for recognizing a neuromere. One is differential gene expression respecting neuromeric boundaries. The EmT expresses genes leading to glutamatergic neuronal development (see: The specifics) and is, thus, genoarchitectonically different from the ventral thalamus which expresses genes involved in GABAergic neuronal development (see: The specifics). This would rather speak for EmT not being part of P3. Differential gene expression may be consistent with recognizing a neuromere, but is not sufficient because many examples exist for intraneuromeric genoarchitectonic differences (see: The specifics). Thus, a second requirement for a neuromere are boundaries and interrelated clonal cell restriction as discussed above for amniote rhombomeres. However, this requirement is also not sufficient because boundaries can arise through other than neuromeric processes, as for example, the pallial-supallial boundary which is neither transverse nor neuromeric. Critical is that a neuromeric boundary extends throughout the dorsoventral extent of the neural tube from roof to floor plate as is clearly the case for rhombomeres. In the zebrafish (as discussed similarly in amniotes above), there is evidence for a forebrain-midbrain boundary [Scholpp et al., 2003; Erickson et al., 2007] and of course there is in zebrafish, as always in vertebrates, a boundary formed by the zona limitans intrathalamica between P2/P3 [Scholpp et al., 2006; Peukert et al., 2011; Mattes et al., 2012; Wullimann and Umeasalugo, 2020]. However, neuromeric boundaries between P1-P2 and P3-Secondary prosencephalon have not been described to my knowledge in any vertebrate. In conclusion, the EmT does not fulfill the requirement for a separate neuromere nor is it evidently part of P3. For the latter to be the case, a continuous neuromeric boundary as defined above would have to be shown anterior to EmT and the rest of P3. Thus, the author has remained with this three-prosomere plus secondary prosencephalon model as an explanatory instrument ever since and it will be used in the following (see: The specifics).

The three-prosomere model subsequently gained strong support by various zebrafish brain developmental gene expression patterns [Hauptmann and Gerster, 2000; Hauptmann et al., 2002; Lauter et al., 2013]. Thus, three prosomeres form the posterior forebrain, including P1 (pretectum), P2 (dorsal thalamus), and P3 (prethalamus, formerly ventral thalamus) from posterior to anterior also in the zebrafish. Equally important for the model is that the posterior forebrain has alar and basal plate components (as does the anteriorly lying hypothalamus/telencephalon or secondary prosencephalon, SePr in Fig. 1h). More details will be discussed in Part 2 (The specifics).

## Part 2: The Specifics

In this second part, the author focuses on three cases in which the neuromeric/prosomeric approach tremendously helped zebrafish forebrain research.

### The Early Migrating Forebrain Areas M1 through M4

The first prominent example where the prosomeric model has been instrumental for resolving the developmental relationship of zebrafish larval to adult structures is how the identity of early migrated teleostean forebrain areas has been resolved. The adult teleostean forebrain contains various migrated nuclei (indicated by red letters in Fig. 2; for all abbreviations see legend). In the zebrafish, these include migrated subpallial nuclei (Vc,Vl; Fig. 2a) followed posteriorly by dorsal and ventral entopeduncular nuclei (ENd, ENv; Fig. 2b). In the rostral diencephalon, the zebrafish pretectum exhibits various migrated nuclei (PSp, PSm, CPN, DAO; Fig. 2d) and, finally, the caudal diencephalon has various migrated nuclei belonging to the preglomerular complex (PGa/PGl/PGm; Fig. 2e; for full account see [Wullimann et al., 1996]; e.g., regarding an alternative hypothesis that the PSp might be homologous to the griseum tectale of birds). When the larval zebrafish brain came into research focus, four early migrated forebrain areas (M1 through M4; orange structures in Fig. 1b) were described at comparable forebrain levels (easily seen in a stain of Hu-proteins for early differentiated neurons; Fig. 3a-d panels modified from [Mueller and Wullimann, 2016]). Although at first sight, a one-to-one relationship between larval M4 through M1 and telencephalic, entopeduncular, preglomerular, and pretectal areas is attractive, the real situation turns out to be more complicated.

**Fig. 4:**
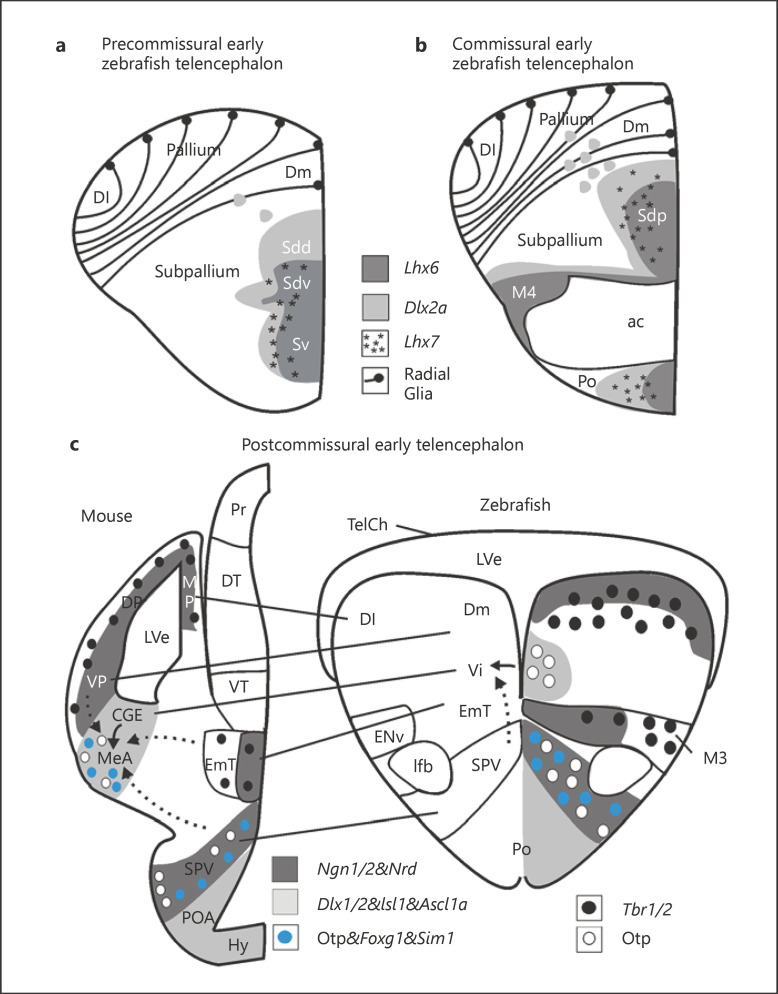
Development of larval zebrafish subpallium. Transverse sections show precommissural (**a**) and commissural (**b**) telencephalon, as well as medial amygdala and eminentia thalami in early mouse and zebrafish postcommissural telencephalon (**c**) with critical gene expression [modified from Gerlach and Wullimann, 2021; see there for more references on mouse and zebrafish gene expression]. Solid arrows designate radial migrations, dotted arrows designate tangential migrations. ac, anterior commissure; CGE, caudal ganglionic eminence; Dl, lateral zone of dorsal telencephalic area; Dm, medial zone of dorsal telencephalic area; DP, dorsal pallium (isocortex); DT, dorsal thalamus; ENv, ventral entopeduncular nucleus; EmT, eminentia thalami; Hy, hypothalamus; lfb, lateral forebrain bundle; LVe, lateral (telencephalic) ventricle; M3, early larval migration zone of eminentia thalami (= ENv); M4, early larval telencephalic migration zone (subpallial); MeA, medial amygdala; MP, medial pallium; Po, preoptic area (zebra­fish); POA, anterior preoptic area (mouse); Pr, pretectum; Sd, larval dorsal part of subpallium; Sdd, dorsal subdivision of Sd (striatum homomog); Sdv, ventral subdivision of Sd (pallidum homolog); Sdp, posterior subdivision of Sd (subpallial amygdala homolog); SPV, supraopto-paraventricular region; Sv, larval ventral part of subpallium (septum homolog); TelCh, tela choroidea; Vi, intermediate nucleus of ventral telencephalon (medial amygdala homolog); VP, ventral pallium (pallial amygdala); VT, ventral thalamus (prethalamus). For gene names see text.

Subsequent larval zebrafish brain developmental studies involved expression analysis of basic Helix-Loop-Helix (bHLH) and downstream expressed genes functionally related to neurogenesis and revealed great similarities to amniote telencephalic development (reviewed in [Mueller and Wullimann, 2016]). These zebrafish studies showed that bHLH genes *Neurogenin1* (*Ngn1*) and *NeuroD* are expressed in developing neurons in the zebrafish pallium (Fig. 4c, right panel). In contrast, *Zash1a* (*Ascl1a*, formerly *Mash1* in mouse) is complementarily expressed in the subpallium (review in [Gerlach and Wullimann, 2021]), as is the downstream subpallial marker gene *Dlx2a* (Fig. 4a, b). For the sake of simplicity, respective zebrafish pallial and subpallial expression domains of bHLH genes *Ngn1/NeuroD* and *Ascl1a* are only shown in panel 4c but not in Figure 4a, b (see [Wullimann and Mueller, 2002] and [Mueller and Wullimann, 2002b, 2003] for full account). The LIM genes *Lhx7* and *Lhx6* are more restrictively expressed in the pallidal part of the subpallium (i.e., Sdv), and *Lxh6* extends − like *Dlx2a* − laterally into peripherally migrated cell masses of **M4** (Fig. 3a1; Fig. 4 a, b). Furthermore, *gad67* (*gad1b*), the gene coding for the synthetic enzyme leading to GABA, and the latter itself (both not shown) have fitting larval subpallial expression pattern (not shown; see [Mueller et al., 2006, 2008]). Thus, the message regarding the identity of M4 could not be clearer: these larval migrated cell masses express genes diagnostic for developing inhibitory GA­BAergic neurons ([Mueller et al., 2008]; more literature summarized in [Mueller and Wullimann, 2016]) which are finally seen in the adult migrated subpallial nuclei (Vc, Vl; [Mueller and Guo, 2009]), as also in the non-migrated (periventricular) subpallial nuclei (Vv,Vd). However, also the adult dorsal entopeduncular nucleus (ENd) is GABAergic [Mueller and Guo, 2009] which identifies it as another (i.e., subpallial) derivative of M4. Indeed, later fate studies confirmed that (pallidal) Vl, Vc, and ENd (but not ENv) derive from various *Dlx* gene expressing cells [Solek et al., 2017].

The situation is completely different for the adult zebrafish ventral entopeduncular nucleus (ENv; Fig. 2b). For starters, it does not contain GABA cells [Mueller and Guo, 2009]. This is in line with larval peripherally migrated cell mass of the eminentia thalami **M3** being characterized genoarchitectonically by expression of bHLH genes and downstream expressed genes that are diagnostic for excitatory glutamatergic neuronal development, i.e., the bHLH genes *Ngn1* and *NeuroD* ([Wullimann and Mueller, 2004a]; summarized in [Mueller and Wullimann, 2016]; Fig. 3e) and in particular by expression of *Tbr1* [Mione et al., 2001] and *Tbr2* genes [Mueller et al., 2008]. In contrast, the eminentia thalami lacks gene expression leading to the GABAergic neuronal phenotype (i.e., *Ascl1a*, *Dlx1/2* or for that matter, *gad67* [Mueller et al., 2008]). Thus, the zebrafish eminentia thalami and its derivates are glutamatergic. In particular the expression of *Tbr1* and *Tbr2* genes (which are expressed in pallium of both zebrafish and amniotes) in the migrated area M3 identifies the latter comparatively as a derivative of the eminentia thalami (which in the mouse expresses both *Tbr* genes [Englund et al., 2005]). Thus, the identification of ENv as a derivative of the eminentia thalami is supported by comparable developmental gene expression data in mouse (review in [Gerlach and Wullimann, 2021]). This led to the conclusion that whereas the dorsal entopeduncular nucleus (ENd) is part of the subpallium (pallidum), the ventral entopenduncular nucleus (ENv) is a derivative of the eminentia thalami and likely homologous to the bed nucleus of the stria medullaris [BNSM; Mueller and Guo, 2009; Mueller and Wullimann, 2009], a conclusion also reached after similar experiments by Ganzet al. [2012].

This identification was then confirmed by the demonstration that the zebrafish ENv forms a major projection to the habenula [Hendricks and Jesuthasan, 2007; Turner et al., 2016] expected for the BNSM. Furthermore, Turner et al. [2016] used zebrafish *Lhx5*-GFP and *Lhx5*-Kaede transgenic zebrafish lines to corroborate the habenular input from ENv and to show elegantly the origin of the ENv from the embryonic eminentia thalami, respectively. This paper also corroborated that ENv cells express *Tbr1,* are calretinin positive and glutamatergic [Turner et al., 2016]. These authors concluded that the zebrafish ENv is a small glutamatergic part of the pallidum (otherwise known to consist overwhelmingly of GABAergic cells), as had been done before Amo et al. [2014], a conclusion not followed here for the following reasons. The mammalian (e.g., rodent) entopeduncular nucleus corresponds to the primate internal globus pallidus, hence the historical speculative naming of teleostean End/ENv. Clearly, the rodent entopeduncular nucleus (i.e., internal pallidum) forms a major input to the lateral habenula [Batalla et al., 2017; Fakhoury, 2018; Roman et al., 2020]. Furthermore, somewhat controversial cellular co-release of GABA and glutamate has been reported in this pathway [Shabel et al., 2012, 2014]. However, in the adult zebrafish brain, only ENd contains GABAergic neurons [Mueller and Guo, 2009], qualifying ENd as part of pallidum, but it does not project to the habenula [Turner et al., 2016]. In contrast, the adult ENv has no GABA neurons [Mueller and Guo, 2009] but does project to the habenula [Hendricks and Jesuthasan, 2007; Turner et al., 2016]. Moreover, there is an alternative comparative interpretation for the ENv because mammals have a bed nucleus of the stria medullaris (BNSM) whose cells are enkephalinergic and project to the medial habenula ([Shinoda and Tohyama, 1987; Risold and Swanson, 1995]; recent review by [Roman et al., 2020]). Indeed, Abbott and Jacobowitz [1999] describe the developing mouse eminentia thalami as transiently calretinin positive cells which massively contribute axons to the stria medullaris leading into the habenula. This is highly similar to what is described above for the zebrafish ENv [Hendricks and Jesuthasan, 2007; Turner et al., 2016]. Thus, development, molecular neurogenetics, and adult neuronal markers as well as connectivity allow for the teleostean M3/ENv to alternatively correspond to the mammalian BNSM and not to part of the pallidum.

A further comment is necessary regarding the most caudal (postcommissural) telencephalic level (Fig. 4c). We had only recently discussed comparatively the larval zebrafish and embryonic mouse forebrain [Gerlach and Wullimann, 2021]. Briefly, the zebrafish subpallium includes septal (Sv = adult Vv), striatal (Sdd = adult Vdd), pallidal (Sdv = adult Vdd) and subpallial amygdalar (Sdp = adult Vs, Vp,Vi) divisions (Fig. 2a, b; Fig. 3a-d, Fig. 4a, b, for abbreviations see respective legends). The entire larval subpallium is characterized by gene expression associated with the developing GABAergic neuronal phenotype, such as the bHLH gene *Ascl1a* (formerly *Zash1a* [Wullimann and Mueller, 2002]) or various *Dlx* genes (*Dlx1a* [Zerucha et al., 2000]; *Dlx2a* [Mueller et al., 2008]; *Dlx5/6* [Turner et al., 2016]:). The most posterior subpallial area (i.e., the intermediate nucleus of ventral telencephalon, Vi, was most recently characterized in larval and adult zebrafish brains [Herget et al., 2014; Biechl et al., 2017] and recognized as the homolog of the amniote medial amygdala (Fig. 4c). This was based on kin recognition related behavior, vomeronasal-like peripheral input, higher-order neuronal connectivity, and neuronal activity (review by [Gerlach and Wullimann, 2021]).

**Fig. 5:**
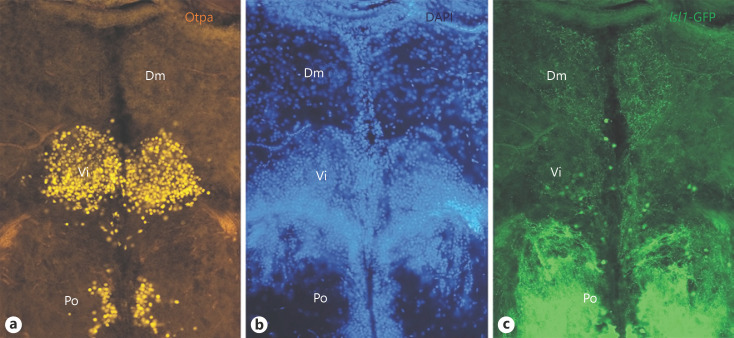
Transverse sections through the zebrafish most caudal amygdalar formation, shows the intermediate nucleus of ventral telencephalon (Vi), the homolog of the medial amygdala. **a** Otpa, DAPI (**b**) and *Islet1*-GFP (**c**). Note fine *Islet1*-positive terminals in medial zone of dorsal telencephalon (Dm). See text for details. Abbreviations: Dm, medial zone of dorsal telencephalon, Po, preoptic region.

Moreover, Vi cells contain orthopedia (Otp) protein (Fig. 5a), a diagnostic transcription factor for the medial amygdala [Herget et al., 2014; Affaticati et al., 2015; Biechl et al., 2017]; see also Section 2). Furthermore, the transcription factor coding gene *islet1* is expressed basally along the neuraxis (zebrafish [Higashijima et al., 2000; Baeuml et al., 2019]). *Islet1* was reported to be expressed in subpallial Vv/Vd/Vs (the latter two only partly), but not in the caudal subpallial divisions (Vp/Vi), although a distinct *Islet1*-GFP terminal field was erroneously reported in Vi (Fig. 3c-c″; of [Baeuml et al., 2019]). However, a re-examination of zebrafish *Islet1*-GFP brains counterstained with Otp antibody showed that the Otp positive cell bodies of Vi are ventral to this terminal field, the latter being itself in the caudal medial zone of the pallium (Dm). Moreover, a few *Islet1*-positive cells are present in Vi (Fig. 5b).

Porter and Mueller [2020] recently reported based on the *Lhx5*-GFP line (already discussed above in the context of the eminentia thalami) and Otp immunohistochemistry that also Vi expresses *Lhx5*, cellularly co-localized with Otp. Because the eminentia thalami and its derivative, the ENv are characterized by *Lhx5* expression [Turner et al., 2016], Porter and Mueller [2020] concluded that Vi is another (i.e., rostral) division of the eminentia thalami. The author disagrees strongly with this conclusion. First, the zebrafish preoptic region also contains *Lhx5* cells and, thus, one could equally argue that Vi is a part of the preoptic region (see Section 2). Second, it has been convincingly shown that the amniote medial amygdala (see citations and discussion in [Gerlach and Wullimann, 2021]) is a basically GABAergic division of the subpallial amygdala which receives unusual large contributions of glutamatergic cells from other regions via tangential migration (Fig. 4c; dotted arrows in left panel). These include for example *Lhx9* positive cells from the ventral pallium [García-López et al., 2008; Bupesh et al., 2011b] or − important for the argument here − Otp/*Lhx5* positive cells from the supraopto-paraventricular (preoptic) region ([García-Moreno et al., 2010]; see Section 2), plus − somewhat ironically − *Pax6* positive cells from the eminentia thalami [Puelles et al., 2000; Abellán and Medina, 2009; Bupesh et al., 2011a]. Thus, the Otp/*Lhx5* cells in the amniote medial amygdala are not born in the subpallium but rather arrive there from the SPV. Thirdly, whereas these invading cells are excitatory glutamatergic cells, there is no question that the amniote medial amygdala also contains GABAergic cells (most recently nicely documented by [Morales et al., 2021]). These GABA cells are the autochthonously and radially generated subpallial cells of the medial amygdala that identify it as a subpallial structure, while the numerous additional glutamatergic cells are from extraneous sources. There is no compelling evidence in Porter and Mueller [2020] to show that the situation is different in the zebrafish brain. The author considers the teleostean Vi as a subpallial, basically GABAergic structure. Indeed Porter and Mueller [2020] do mark Vi/MeAp as GABAergic and *gad67* positive (in their Table 1). Of course, the teleostean Vi is supplemented with numerous and varied glutamatergic cells having invaded Vi from other origins, such as the SPV (see Section 2) as is the case in amniotes. Of note, Pax6 protein is expressed periventricularly in the zebrafish eminentia thalami (not yet designated as such, but shown in Fig. 2c at upper rim of the preoptic region (Po) in [Wullimann and Rink, 2001]) but, apparently, no Pax6 positive cells migrate into the Vi.

The early migrating area **M2** (Fig. 1b) lies in the lateral periphery of the larval zebrafish posterior tuberculum and extends from rostral (Fig. 3c) to caudal (Fig. 3d) diencephalic levels. The larval posterior tuberculum includes two proliferation zones (PTd, PTv; Fig. 1g) which represent the basal plate parts of P2/P3. The cell masses M2 are notable because they have unusual ongoing proliferative activity far away from their ventricular origin [Mueller and Wullimann, 2002a]. There is no reasonable doubt that the larval M2 cell masses give rise to the so-called preglomerular complex (PG) consisting of various prominent adult nuclei in teleosts (zebrafish: see Fig. 2e) that are species-specifically enlarged depending on the relative predominance of particular sensory systems. The preglomerular nuclei have distinct sensory, i.e., auditory, lateral line, somatosensory, gustatory or visual, representations and relay this information coming from ascending pathways to the telencephalon (case studies in the sea ruffe [Murakami et al., 1986]; elephant-nose fish [Prechtl et al., 1998; von der Emde and Prechtl, 1999]; goldfish; rainbow trout [Folgueira et al., 2005; Northcutt, 2006]; reviews [Wullimann and Mueller, 2004b; Vernier and Wullimann, 2009]). Nevertheless, the teleostean preglomerular complex has remained somewhat enigmatic. Although its sensory relay function resembles functionally the amniote dorsal thalamus, the origin of PG is clearly not from the dorsal thalamic proliferation zone/histogenetic unit [Wullimann, 2020]. The teleostean dorsal thalamic nuclei (A, DP, CP, Fig. 2d, e) are indeed also related to relaying sensory (i.e., auditory/visual) information to the telencephalon, but they do so mostly to the subpallium [Northcutt, 2006]. In contrast, the PG has massive reciprocal interconnections with pallial divisions (review [Vernier and Wullimann, 2009]). The author has recently summarized the various embryonic cellular origins of the M2/PG that have historically been postulated [Wullimann, 2020]. This clarified that the cell contributions arising from the posterior tuberculum [Wullimann and Umeasalugo, 2020] and maybe from ventral thalamus [Wullimann and Rink, 2001; Ishikawa et al., 2007] migrate radially into M2, whereas additional large contributions from the alar midbrain [Bloch et al., 2019, 2020] do so tangentially. Many (or maybe all) PG cells arising in the posterior tubercular ventricular zone express *sonic hedgehog* ([Wullimann and Umeasalugo, 2020]; see also Section 3) and are likely glutamatergic whereas those from the ventral thalamus express *Dlx2* and *Pax6* [Wullimann, 2020] and are thus highly likely GABAergic. The latter are a small fraction of the adult PG [Mueller and Guo, 2009]. This research establishes the teleostean PG as a derivative of multiple sources. However, since the radial glia as a “natural coordinate system” [Nieuwenhuys, 1998] extending from a generative ventricular zone into its peripherally migrated area is decisive for the latter's primary neuroanatomical assignation, the PG is interpreted as being primarily a posterior tubercular structure arising from basal plate ventricular zones of P2/P3 [Wullimann, 2020; Wullimann and Umeasalugo, 2020].

Finally, the most posterior migrated zebrafish forebrain area **M1** is within the pretectal histogenetic unit P1 (Fig. 1b; Fig. 3d). The pretectum can genoarchitectonically be well differentiated from the anteriorly adjacent dorsal thalamus (Fig. 3e, modified from [Wullimann and Mueller, 2004a; Mueller and Wullimann, 2016]). In addition to having uniquely shaped expression domains of *Pax6*, *Ngn1*, and *NeuroD*, the pretectum also expresses broadly *Ascl1a* (formerly *Zash1a*; Fig. 3e). In contrast, *Ascl1a* is expressed within the dorsal thalamus only very anteriorly and close to the zona limitans intrathalamica (shown in Fig. 14 in [Mueller and Wullimann, 2016]). The zebrafish pretectum has furthermore − unlike the dorsal thalamus − large serotoninergic (zebrafish [Kaslin and Panula, 2001; Rink and Guo, 2004]; also in various other teleosts [Rosner et al., 2019]) and dopaminergic cell clusters [Kaslin and Panula, 2001; Rink and Wullimann, 2001; Yamamoto et al., 2011; Kress and Wullimann, 2012]. The adult zebrafish brain exhibits apart from the periventricular pretectum (PPd/PPv; Fig. 2e) additional migrated nuclei, including a central (CPN) and two superficial pretectal nuclei (PSm, PSp), as well as the dorsal accessory optic nucleus (DAO) (Fig. 2d). All these nuclei, except the PSm, are retinorecipient [Northcutt and Wullimann, 1988]. We have recently used the online mapzebrain zebrafish brain atlas of the Baier laboratory to study the location of diencephalic cells with dendrites into particular larval retinal terminal fields [Baier and Wullimann, 2021]. Regarding the pretectum, we found that such cells functionally characterized as direction-sensitive or related to prey-catching in the zebrafish larva (which would correspond to adult CPN-DAO or PSp, respectively), are all within the larval M1 cell masses This speaks strongly for the interpretation that M1 gives rise to all adult pretectal (incl. accessory optic) nuclei and is therefore a migrated zone of pretectal prosomere P1.

### Alar (Preoptic) Hypothalamus and Basal Hypothalamus

The vertebrate **preoptic region** is traditionally considered an anterior part of the hypothalamus. Later prosomeric models recognize it as part of the subpallium [Flames et al., 2007; Abellán and Medina, 2009]. We discussed previously in detail the somewhat different (i.e., more inclusive) use of the term *preoptic region* in teleosts (e.g., zebrafish) as compared to amniotes ([Herget et al., 2014]; compare Fig. 1a, b). The larval teleostean (e.g., zebrafish) preoptic region is a large alar area of the secondary prosencephalon between anterior and postoptic commissures bordered by subpallium (S), eminentia thalami (EmT), ventral thalamus (VT) and basal hypothalamus (H, separated by a chain line from alar plate hypothalamic regions; Fig. 1b). In the adult teleostean (e.g., zebrafish) brain, the preoptic region includes a magnocellular (including a gigantocellular portion) and an anterior and posterior parvocellular preoptic nucleus as well as the suprachiasmatic nucleus which together represent the entire **alar hypothalamus** (Fig. 2b-d i [Wullimann et al., 1996; Herget et al., 2014]).

Thus, the zebrafish preoptic region represents the alar part of the hypothalamus and was shown to contain the so-called supraopto-paraventricular region (SPV; Fig. 1b; Fig. 3e; Fig. 4c). In rodents, the SPV is different genoarchitectonically from the remainder of the preoptic region (reviewed in [Osório et al., 2010]; see also Section 1). For example in the mouse, part of the preoptic region expresses genes involved in the GABAergic neurogenetic pathway, whereas the SPV expressess markers of glutamatergic cell development, such as the bHLH genes *Ngn2* and *NeuroD* [Osório et al., 2010], the LIM homeodomain gene *Lhx9* [Rétaux et al., 1999; García-López et al., 2008], and, importantly, the *Orthopedia* (*Otp*) gene is typically expressed in the SPV ([Bardet et al., 2008]; see Fig. 4c). The rodent SPV contains the adult paraventricular and supraoptic nuclei which represent the cellular loci of neuroendocrine neuropeptides including oxytocin (formerly isotocin in teleosts) and vasopressin (formerly arginin vasotocin in teleosts [Theofanopoulou et al., 2021] and various additional neurosecretory peptides (releasing and inhibiting factors) which all additionally act via axons as CNS neurotransmitters ([Swanson and Sawchenko, 1983; Ferguson et al., 2008; Simmons and Swanson, 2009]; see [Herget et al., 2014] for discussion). Thus, the amniote SVP may be seen as a glutamatergic preoptic/hypothalamic domain which contains the core nucleus of the stress-regulatory axis, the nucleus paraventricularis. In contrast, preoptic cells directly bordering the SPV and also those in subpallium, prethalamus and basal hypothalamus express genes indicative for developing GA­BAergic cells, such as *Ascl1*, *Dlx5*, *Arx*, and *Islet1* (reviewed for amniotes in Herget al., 2014; see also next section).

In the adult zebrafish, the magnocellular preoptic (PM, incl. the gigantocellular part PMg; Fig. 2c, i) and the neurosecretory part of the posterior parvocellular preoptic nucleus (PPp; Fig. 2c, b, i) were homologized with the amniote paraventricular and supraoptic nuclei, respectively [Herget et al., 2014]. Apart from Otp (transcription factor) and oxytocin/vasopressin immunohistochemistry in the adult zebrafish brain, this conclusion was based on a detailed developmental description of various additional neuropeptides within the SPV and expression of transcription factors *Dlx5*, *Arx* and *Islet1* in the remainder of the zebrafish preoptic region and beyond (i.e., in subpallium, ventral thalamus and basal hypothalamus) for delineating the genoarchitecture of the SPV and surrounding regions [Herget et al., 2014]. Furthermore, in line with these larval data, *Islet1* is present in anterior/posterior parvocellular preoptic nuclei in the adult zebrafish, but only a few dispersed *Islet1*-positive cells are present in (i.e., likely invaded) the magnocellular preoptic nucleus [Baeuml et al., 2019]. Moreover, Affaticati et al. [2015] described complementarily the zebrafish SPV as being located at the lateral tip of the preoptic recess surrounded by adjacent *Dlx2a* expression (similar to *Dlx5* as described above), the SPV itself being glutamatergic as evidenced by expression of bHLH gene *Neurogenin1*. These authors furthermore divided the SPV molecularly into an anterior *Sim1a/Foxg1* positive domain (overlapping with *otp*) and a posterior *Sim1a*-only expressing domain, as similarly seen in mouse (Fig. 4c; [Morales et al., 2021]; reviewed in [Gerlach and Wullimann, 2021]).

As just mentioned, the zebrafish so-called preoptic region is more inclusive than the amniote areas designated as such (i.e., POA) which are only a part of the alar hypothalamus (compare Fig. 1a, b). However, beyond what is described above for the zebrafish, the present genoarchitectonic resolution of the zebrafish preoptic region does not allow to further relate the latter in more detail to particular amniote alar hypothalamic subdivisions which have been updated greatly [Díaz et al., 2015]. The term SPV is no longer used [Díaz et al., 2015]. However, the paraventricular nucleus or area, as expected, is contained in the (paraventricular) glutamatergic alar hypothalamus (which has six divisions and genes expressed include *Otp*, *Sim1*, and *vGlut2*). The (subparaventricular) GABAergic alar hypothalamus instead has two divisions and its genetic markers include *Gad67*, *Dlx*, *Arx*, and *Islet1* [Díaz et al., 2015]). Moreover, an admirably detailed analysis of source areas of various neuropeptides (e.g., oxytocin and vasopressin are generated only in glutamatergic alar hypothalamus, but preproenkephalin and galanin additionally in basal hypothalamus) and subsequent radial and tangential migrations within the entire (including basal) hypothalamus are suggested to occur [Díaz et al., 2015].

This corresponds overall well to the picture described for the glutamatergic SPV and the GABAergic remaining preoptic region in the zebrafish brain above. However, a formerly *preoptic* area in the mouse alar hypothalamus (also expressing *Ascl1*, *Dlx*, *Arx* and *Islet1*) towards to the subpallium is excluded from it and newly assigned to the subpallium [Díaz et al., 2015]. This area corresponds to the zebrafish anterior parvocellular preoptic nucleus (PPa; Fig. 2b, i) and is clearly separate from subpallial ventral telencephalic areas (Vv,Vd; Fig. 2a, i; note position of anterior commissure in Fig. 2i). Moreover, the teleostean PPa is particularly rich in neuropeptidergic galanin-positive cells (e.g., goldfish [Rao et al., 1996]; plainfin midshipman [Tripp and Bass, 2020], see there for many more citations on teleosts). Furthermore, in the midshipman, many galanin-positive PPa cells co-express GABA and have projections to the subpallium (Vd,Vv), whereas only few galanin cells are seen in PM and PPp [Tripp and Bass, 2020]. The PPa galanin-positive cells are often dimorphic between sexes (e.g., red salmon [Jadhao and Meyer, 2000]; sailfin molly [Cornbrooks and Parsons, 1991]; midshipman [Tripp and Bass, 2020]) and between males with different mating strategies (e.g. [Tripp et al., 2020]). The PPa is therefore similar to the mammalian galanin-/GABAergic embryonic POA (see Fig. 1a) and the adult rodent medial preoptic nucleus [Wu et al., 2014]. For this bouquet of reasons, the author does not consider the teleostean PPa and its larval primordium as subpallial, because it is morphologically, developmentally and functionally cleary identifiable as part of the GABAergic alar (preoptic) hypothalamus. Of note, the terminology shown here in the schema for the mouse (Fig. 1a) is from the original paper by Puelles and Rubenstein [1993] because this explanatory schema is used in several of our and other publications discussed here. Thus, the author keeps this schema here in order to relate intelligibly and lucidly to this previous literature.

Similarly, the amniote **basal hypothalamus** at that time had been subdivided anteroposteriorly in a retrochiasmatic (RCH), a tuberal (TU), and a mammillary (MA) region (Fig. 1a; showing an early version of the prosomeric model), but has been equally greatly revised and refined since [Puelles et al., 2012; Ferran et al., 2015]. The author discusses now relevant teleostean (in particular zebrafish) data on basal hypothalamus with respect to these novel mouse analyses.

A reasonable starting point is to consider the morphological divisions of the larval [Mueller and Wullimann, 2016] and adult zebrafish basal hypothalamus [Wullimann et al., 1996]. Continuing with transverse adult zebrafish brain sections beyond the subpallium (Vd/Vv, Fig. 2a) and preoptic region (PPa, Fig. 2b, c), one enters, at the level of the postoptic commissure, the basal hypothalamic cell masses (H; Fig. 1b, Hv; Fig. 2d). This part of the teleostean basal hypothalamus was historically called ventral hypothalamic periventricular zone (Hv; e.g., in the zebrafish, Fig. 2d). In contrast to the just described teleostean preoptic region, the Hv has no ventricular recess. At the next transverse level, the hypothalamic ventricle shows a lateral recess (LR; Fig. 2e) and the cell masses surrounding it were designated dorsal zone of periventricular hypothalamus (Hd, note its apparent “dorsal” position relative to Hv). These two adult hypothalamic divisions (Hv/Hd) are characterized by distinct nuclei, such as the anterior tuberal nucleus (ATN) and the lateral hypothalamic nucleus (LH; Fig. 2e). Peripherally, various migrated nuclei of the inferior lobe (see below) are seen (e.g., DiI, Fig. 2e-g). The remarkable extension of Hd is reflected in the fact that it bends caudoventrally and forms the so-called inferior lobe which carries within it the lateral recess ventricle (LR; Fig. 2e-h). Thus, the inferior lobe (LI; Fig. 1f) (containing the tip of Hd) lies at this position lateral to the third basal hypothalamic division, which is the caudal zone of the periventricular hypothalamus (Hc). The Hc exhibits a (third) ventricular hypothalamic recess, the posterior recess (PR; Fig. 2g). At this level, the bilateral periventricular cells masses of Hc are accompanied by midline cells that belong to Hc which are contiguous with the posterior tuberal nucleus (PTN; Fig. 2g). Also, the conspicuous so-called corpus mamillare (CM, mammillary body) lies at this adult hypothalamic level.

Later, we brought up the conflict between general vertebrate body axes versus neuraxes which is due to the deflection of the vertebrate anterior neuraxis relative to the general body axis during development (see Fig. 1h; after [Herget et al., 2014]). Thus, we suggested to use along the neuraxis the terms anterior/posterior and alar/basal (i.e., true dorsal/ventral for the neural tube). Instead, rostral/caudal (i.e., towards the rostrum/tail) and dorsal/ventral (i.e., towards the back/belly) should be used for general body axes. In this way, if interpreted according to the zebrafish anteroposterior neuraxis (red dotted line in Fig. 1h), transverse sections of the basal hypothalamus in the general body anteroposterior direction starting from the anterior tip of the brain (oc/poc) in fact run from dorsal (d) to ventral (v) (compare gray area in Fig. 1h). Realizing these axes relationships, we used in our developmental studies in the larval zebrafish brain the term rostral hypothalamic periventricular zone (Hr) according to the general body axis (corresponding to the adult Hv; reviewed in [Mueller and Wullimann, 2016]). The larval zebrafish Hr is followed caudally by the intermediate (Hi, i.e., the adult Hd) and then by the caudal hypothalamic periventricular zone (Hc), both of which are identified already early by exhibiting a discreet lateral (LR; Fig. 3d, f) and posterior ventricular recess (PR; Fig. 3f), respectively. Thus, the designations used for the larval zebrafish hypothalamus (Hr/Hi/Hc) follow the general vertebrate rostrocaudal body axis (compare Fig. 1g with h). Importantly, already in the larva, the inferior lobe containing Hi (and LR) is bent caudoventrally and lies lateral to Hc (Fig. 3f). These morphological relationships make clear that the LI is part of the intermediate hypothalamus and lies topologically (i.e., according to the neuraxes) more dorsal than Hc. Thus, rather than being misleading as interpreted by Schredelseker and Driever [2020], these three larval hypothalamic divisions (Hr/Hi/Hc, see Fig. 1g) follow the clearly defined rostrocaudal general body axis and provide a handle to understand teleostean hypothalamic morphology.

Regarding bHLH transcription factors in the zebrafish larval basal hypothalamus (Fig. 1i), we reported extensive *Ascl1a* (formerly *Zash1a*) − but no *Neurogenin1* − expression in the basal hypothalamus; expression of *NeuroD* was only seen in the hypophysis [Wullimann and Mueller, 2002; Mueller and Wullimann, 2003]. Furthermore, the larval zebrafish basal hypothalamus broadly expresses various *Dlx* genes and *Gad67* [Mueller et al., 2008; MacDonald et al., 2010]. This is in line with the finding that GABA positive cells are found peripherally in all zebra­fish larval hypothalamic periventricular cell masses (Hr/Hi/Hc [Mueller et al., 2006]), suggesting that the basal hypothalamus contains many GABAergic cells. Similarly, the adult goldfish basal hypothalamus expresses broadly *Gad67/Gad65* [Martinoli et al. 1990; Martyniuk et al., 2007]. In contrast, *neurogenin1* is not expressed in all three hypothalamic divisions (Hr, Hi, Hc [Mueller and Wullimann, 2003]). However, *neurogenin3* shows some isolated spots of expression in the rostral to intermediate zebrafish basal hypothalamus (Fig. 1i), while *neurogenin2* does not exist in zebrafish (see [Wang et al., 2001]). These authors report a cluster of *Ngn1* in the “mammillary” hypothalamus, interpreted as posterior tuberculum in line with our own data ([Mueller and Wullimann, 2003]; see next section).

These zebrafish data are in accord with our *Ascl1* (formerly *Mash1*)*, Ngn2*, and *NeuroD* embryonic mouse brain expression study and comprehensive review therein of the mouse literature on bHLH gene expression ([Osório et al., 2010]; see there for rodent citations). Thus, in the E12.5 mouse brain basal hypothalamus, there is broad expression of *Ascl1* and GABA/GAD, whereas *Ngn3* is only present co-extensively with *NeuroD* in one particular tuberal area, and both *Ngn1* and *Ngn2* are not expressed in the mouse basal hypothalamus (reviewed in [Osório et al., 2010]). Like in the zebrafish, all this speaks for the mouse basal hypothalamus to contain mainly (autochthonously generated) GABAergic neurons. Of course this does not preclude later invasion of glutamatergic cells into basal hypothalamus neither in mouse nor zebrafish (see for example above for migrating glutamatergic-neuropeptidergic cells from SPV) [Díaz et al., 2015; Herget and Ryu, 2015]. Furthermore, in the adult mouse brain, glutamatergic (i.e., *vGlut2* positive) cells are abundant in part of tuberal (e.g., ventromedial nucleus) and mammillary hypothalamus (e.g., retromammillary and mammillary nucleus) and their distribution is roughly complementary to that of GABAergic (i.e., *Gad67* positive) cells [Puelles et al., 2012]. It remains unclear to what extent these glutamatergic cells have invaded the basal hypothalamus from extraneous origins or are *Ngn3*-*NeuroD* dependent autochthonically generated cells.

Critical monoaminergic landmarks in the adult teleostean (i.e., zebrafish) basal hypothalamus are firstly histaminergic cells (the sole CNS population) forming an outer rim within the periventricular cell zone surrounding the posterior recess of Hc (posterior paraventricular organ of [Kaslin and Panula, 2001]) accompanied by many serotoninergic (5-hydroxytryptamine, 5-HT) cells, located closer to the posterior recess ventricle [Kaslin and Panula, 2001]). Secondly, another dense cluster of serotoninergic cells is present in the dorsal part of Hd (called intermediate nucleus of Hd [Rink and Wullimann, 2001]; the intermediate paraventricular organ of [Kaslin and Panula, 2001]). Both dominant serotoninergic clusters have recently been shown also in various additional teleostean species [Rosner et al., 2019]. Notably, 5-HT co-localizes with tyrosine hydroxylase 2 (TH2) and dopamine in adult zebrafish Hd and Hc cells (dopamine cell groups 5/6 in Fig. 1e [Vernier and Wullimann, 2009; Yamamoto et al., 2011; Xavier et al., 2017]). Moreover, the zebrafish larva already shows these two serotoninergic/dopaminergic clusters as expected in Hi and Hc ([Rink and Guo, 2004]; Fig. 1i). These hypothalamic serotoninercig/dopaminergic cells are liquor-contacting neurons and such cells occur in all vertebrates (see dopamine cell group in H in *Xenopus*, Fig. 1f) except in placental mammals [Yamamoto et al., 2010; Xavier et al., 2017]. Thus, the so-called “hypothalamic ventricular organ” that is described in placental mammals to accumulate monoamines [Puelles et al., 2012] maybe an evolutionary remnant of the hypothalamic nuclei discussed above for teleosts and are often called “paraventricular” in other vertebrates where, ironically, these neurons synthesize these monoamines (see above and also next section for the zebrafish paraventricular organ in basal P3). Thus, Xavier et al. [2017] argue that in placental mammals no basal hypothalamic dopamine cell group (as A12 in Fig. 1d) is homologous to these liquor-contacting cells of other vertebrates. The loss of these dopamine cell groups is likely related to the parallel loss of tyrosine hydroxylase 2 only in placental mammals within vertebrates [Yamamoto et al., 2010, 2011]. However, non-liquor-contacting dopamine cells have been described in the adult zebrafish lateral hypothalamic nucleus [Rink and Wullimann, 2001; Yamamoto et al., 2011]. Also, mammalian alar hypothalamic A14/15 dopamine groups likely are comparable to dopaminergic teleostean preoptic cell groups.

How do teleostean basal hypothalamic divisions relate to what is known on the mammalian tuberal (medial and lateral) basal hypothalamus, traditionally considered to be anterior to the mammillary hypothalamus (TU/MA in Fig. 1a)? More recent analyses place the mammalian tuberal hypothalamus dorsal to the ventral mammillary one (using the anteroposterior neuraxis [Puelles et al., 2012; Ferran et al., 2015]). These new analyses provide about three dozens of (mostly transcription factor coding) gene expression patterns to characterize the mouse basal hypothalamus genoarchitectonally. Before discussing similar recent zebrafish analyses [Schredelseker and Driever, 2020; Schredelseker et al., 2020], the author considers some earlier established issues.

Generally, the mammalian medial (tuberal) hypothalamus is related to the medial amgydalar output systems via stria terminalis in sociosexual and defensive contexts whereas the lateral hypothalamus relates to central amygdalar output via the ansa lenticularis and is involved among other functions in fear/anxiety and feeding (recently reviewed by [Gerlach and Wullimann, 2021]). Forlano and Cone [2007] elegantly demonstrated in both adult and larval zebrafish that the hypothalamic homeostasis and feeding related melanocortin system, i.e., α-melanocyte-stimulating hormone containing (MSH) cells and antagonistic agouti-related protein (Agrp) containing cells are selectively present in different cell populations of the teleostean (i.e., zebrafish) Hv. Thus, these Hv cells qualify as homolog of the mammalian (amniote) hypothalamic (lateral hypothalamic) arcuate nucleus. Dense projections of both these cell populations in Hv to the preoptic magnocellular nucleus (PM) furthermore support the latter's homology to the mammalian (amniote) nucleus paraventricularis ([Forlano and Cone, 2007]; see above). In the larval zebrafish brain, the Agrp, histamine (using *histidine decarboxylase* expression) and the two 5-HT populations just described for the adult brain were confirmed in the larval zebrafish brain and a corticotropin-releasing factor binding protein (Crhbp) cell population was additionally described in a position where MSH was also found (Fig. 1i; [Schredelseker et al., 2020]).

The intrinsic neuraxes described above (after [Herget et al., 2014]) were then used by Schredelseker and Driever [2020] (their Fig. 1h) and Schredelseker et al. [2020] to clarify various genoarchitectonic issues of the basal zebrafish hypothalamus between 2 and 4 days postfertilization. The two studies describe 4 mammillary and 7 tuberal regions using differential expression of transcription factor (TF) coding homeobox (*Bsx, Arxa, Islet1, Nkx2.1, Nkx2.2, Otpa*), paired-box (*Pax6a, Pax7*), and LIM-homeobox (*Lhx5, Lhx6, Lhx9*) genes, plus distal-less homeobox gene *Dlx5a*, forkhead gene *Foxb1a*, the orphan nuclear receptor gene *Nr5a2,* as well as signaling factor coding genes *Shha* and *Fgf8a.* The authors conclude from these detailed transcription factor gene expression analyses that − similar to the situation in the mouse − the larval zebrafish basal hypothalamus has a large tuberal and a smaller posteroventrally positioned mammillary part (TubH/MamH; Fig. 1h, i). The two signaling factors (*Shha/Fgf8a*) appear to be active in both main basal hypothalamic divisions. The transcription factors *Pax7a* and *Foxb1a* (indicated in red in Fig. 1i) are exclusively expressed in (different) mammillary subregions (but not in TubH), whereas many more TF coding genes are exclusively expressed within subregions of the tuberal basal hypothalamus (but not in MamH) (Fig. 1i). *Lef1*, for example, is focally expressed in the posterior recess region (Hc) and *Nkx2.2a* is complementarily expressed in all but one subregions of TuH. Interestingly, in both larval [Schredelseker and Driever, 2020] and adult zebrafish [Wullimann and Umeasalugo, 2020], the signaling factor *Shha* is absent from the most ventral tuberal area (i.e., the Hc) but present in more dorsal tuberal areas (i.e., adult Hd/Hv). The *Shha* dependent expressed gene *Islet1* is similarly expressed in these tuberal areas in the larval zebrafish brain [Schredelseker and Driever, 2020] but extends more ventrally (i.e., into the Hc area). This is similar in the adult zebrafish brain, where Hc has *Islet1*-positive cells − in addition to such cells in Hd/Hv − in the area just before the posterior recess arises (but not around the recess itself [Baeuml et al., 2019]). Another interesting issue is that *Dlx5a* (as a marker for GABAergic cell development [MacDonald et al., 2010]) is expressed in the entire tuberal hypothalamus except for two *nr5a2* expressing subdivions (i.e., dorsal and intermediate tuberal parts [Schredelseker and Driever, 2020]) and the intermediate tuberal part is identified as homolog of the largely glutamatergic mammalian ventromedial hypothalamic nucleus (see situation in mouse above). *Dlx5a* expression is furthermore absent from the zebrafish mammillary hypothalamus, which also exhibits many glutamatergic cells in the mouse (see above). The author reconsidered our own data and noted in corroboration of these *Dlx5* expression patterns that *Gad67* and *Dlx2a* expression is absent from the region of medial Hc (likely MamH) and in large medial parts of our Hr (part of TubH), but broad expression of these two genes is seen in Hd and Hc around the two respective recesses (see also [Mueller et al., 2008]). Thus, similar to the mouse basal hypothalamus, these medial tuberal and mammillary regions likely contain glutamatergic neurons. In line with these developmental data, the adult zebrafish brain [Mueller and Guo, 2009] shows only most peripherally *Gad67* cells within Hv (including many in ATN), whereas ubiquitous *Gad67* cells sit densely around the lateral recess of Hd (with less scattered *Gad67* positive cells in the diffuse nucleus of the inferior lobe), and Hc is known to express *Gad65* instead [Martyniuk et al., 2007]. Many *Gad67* negative cells of at least the diffuse nucleus originate in tangentially (likely glutamatergic) *Her5* positive cells migrating from the alar midbrain into the basal hypothalamus [Bloch et al., 2019].

Of course, the exact role of all these genes in compartmentalization and cell fate determination of the zebrafish basal hypothalamus must be revealed in future studies. The restriction to optical sagittal and horizontal section material and schematic interpretations offered by this genoarchitectonic larval zebrafish basal hypothalamus analysis [Schredelseker and Driever, 2020] also reveals a few problems. Thus, in sagittal projection analyses, gene expression in Hi lateral to Hc might lead to misinterpretations because the deflected Hi belongs topologically to a more dorsal tuberal division (Fig. 3f; see discussion above). Schredelseker and Driever [2020] furthermore describe that the tuberal hypothalamus includes most of the periventricular cells masses of Hr, Hi, and Hc and that the mammillary hypothalamus includes only the midline region of the posteroventral hypothalamus (Fig. 1h). Thus, the MamH seems to cover for sure the midline of Hc shown in our Figure 3f. Beyond that, the lack of documentation of the third (transverse) section level (as for example, shown in our Fig. 3f) in this (and the previous example) hampers the evaluation of the exact extent of the four mammillary subregions.

### Diencephalic Organization with Emphasis on Dopamine Cells

The prosomeric/neuromeric model has also greatly furthered the understanding of the comparative and functional neurobiology of the basal diencephalon (i.e., basal plate of P1 through P3). Most neurobiologists recognize easily the well-studied alar diencephalic elements, i.e., the large pretectal, dorsal thalamic, and ventral thalamic nuclei of amniotes. Hence, the pretectal, (dorsal) thalamic, and ventral (pre−) thalamic prosomeres P1 through P3 derive their name from these alar diencephalic components. Their respective basal plate portions have not been given equal attention. However, the neuromeric model conception requires that each of the three diencephalic prosomeres has a basal plate part (indicated as bP1, bP2, bP3 for amniotes in Fig. 1a, d; N, PTd, PTv for anamniotes in Fig. 1b, c, e, f; see in [Vernier and Wullimann, 2009; Wullimann, 2020]).

Starting with embryonic mouse and bHLH genes, *Ngn2* shows broad midline expression in basal midbrain and in all three basal diencephalic divisions bP1 through bP3 with a sharp transverse boundary towards the basal hypothalamus (inset in Fig. 1d). Alar pretectum and dorsal − but not ventral − thalamus are also *Ngn2* positive (note in inset that the dorsal thalamus has broken away partly, its real dorsal boundary is indicated by a dashed line). Furthermore, *Ngn2* is expressed in the pallium (P) and the EmT. *NeuroD* is expressed in bP2 through bP3, but *Ngn1* is expressed in bP3 only (see literature summary in Table 1 of [Osório et al., 2010]). However, also *Ascl1* (formerly *Mash1*) is additionally expressed more peripherally in all three basal diencephalic divisions while GABA/GAD is only reported in bP1/2 [see literature summary in Osório et al., 2010]. From these data it seems that the mouse bP1/bP2 generate both GABAergic as well as glutamatergic neurons, and bP3 generates mostly glutamatergic cells.

*Neurogenin1* (*Ngn1*) and its downstream expressed gene *NeuroD* − both marker bHLH genes for glutamatergic cell development − are heavily expressed in the early larval zebrafish brain in alar pretectum (i.e., P1), epiphysis, habenula, and alar dorsal thalamus (i.e., P2) but not in alar ventral thalamus (P3) (Fig. 3e; [Mueller and Wullimann, 2003]). In addition, both genes are expressed in basal areas PTd (bP2) and PTv (bP3), the latter having particularly large expression domains most ventrally ([Mueller and Wullimann, 2003]; see their Fig. 3d2, e2; Fig. 5i). Furthermore, *Ascl1a* (formerly *Zash1a*) − a marker bHLH gene for GABAergic development − is also expressed in the larval zebrafish posterior tuberculum peripherally to *Ngn1*/*NeuroD* ([Wullimann and Mueller, 2002]; see their Fig. 1f2). A reconsideration of our data on larval zebrafish basal diencepalic *Dlx2* and *Gad67* expression shows broad expression of both genes in the posterior tuberculum (PTd/PTv). This is in line with GABA positive cells that lie peripherally in the entire larval zebrafish basal diencephalon throughout bP1 (N), bP2 (PTd), and into bP3 (PTv) [Mueller et al., 2006]. Altogether, these data indicate that both glutamatergic and GABAergic neurons are generated in temporal succession in the zebrafish posterior tuberculum (basal diencephalon). *Ascl1a* is furthermore expressed in alar pretectum (which thus also gives rise to both glutamatergic and GABAergic neurons), in the entire alar ventral thalamus and in only a small area close to the zona limitans intrathalamica in alar dorsal thalamus (see Fig. 14 in [Mueller and Wullimann, 2016]). Adult derivatives of the posterior tuberculum are as follows: the nucleus of medial longitudinal fascicle is considered a derivative of bP1 (N) whereas bP2/bP3 (PTd/PTv) give rise to the periventricular nucleus of the posterior tuberculum (TPp), the paraventricular organ (PVO), and the posterior tuberal nucleus PTN (Fig. 2).

We described adult and larval zebrafish brain dopamine systems comparatively in various publications (Fig. 1d-f [Rink and Wullimann, 2001, 2002; Vernier and Wullimann, 2009; Yamamoto et al., 2010, 2011]). Briefly, the larval zebrafish brain has dopaminergic cell populations in the olfactory bulb (OB), subpallium (S), preoptic region (Po), alar pretectum (Pr), and ventral thalamus (VT, zona incerta/A13 homolog; note that magenta blob 1 at alar-basal boundary in Fig. 1e extends into VT) all of which are not liquor contacting (LQ) [Rink and Wullimann, 2002]. Two zebrafish basal hypothalamic dopaminergic/serotoninergic LQ populations have been discussed above (dopamine populations 5/6 in Fig. 1e). A third dopaminergic/serotoninergic LQ population, the paraventricular organ (PVO, magenta blob 3 in Fig. 1e [Rink and Wullimann, 2001, 2002]; anterior paraventricular organ of [Kaslin and Panula, 2001]) lies in basal plate ventral thalamus (ventral part of posterior tuberculum; Fig. 1e) and is a general teleostean feature (see discussion in [Rosner et al., 2019]). The PVO also exists in amphibians ([Xavier et al., 2017]; magenta blob at PTv/H boundary in Fig. 1f) but is absent− as are all LQ dopaminergic cells (see above) − in placental mammals (Fig. 1d).

However, the zebrafish basal plate ventral thalamus exhibits additional dopaminergic cell populations that are not liquor contacting but instead are either small round neurons (magenta blobs 1 and 7) or large pear-shaped neurons (magenta blobs 2/4 [Rink and Wullimann, 2002]). Small round neurons are found in the adult zebrafish periventricular posterior tubercular nucleus (TPp; larval magenta blob 1) and in the posterior tuberal nucleus (larval magenta blob 7), whereas large pear-shaped neurons (larval magenta blobs 2/4) lie somewhat migrated within the adult TPp. Some zebrafish adult dopamine nuclei mentioned here are depicted in Figure 2.

In the following, the author will focus on dopaminergic ascending projection cells in the basal diencephalon (bP1-bP3; reviewed in [Vernier and Wullimann, 2009]). Agustin González and colleagues were generally instrumental in establishing a new view of early sarcopterygian/tetrapod brain organization. For example, their work demonstrated basal ganglia and amygdalar circuitry as well as the neurochemical organization of modulatory systems to be largely comparable among all tetrapods (e.g., [González et al., 1994a, b; González and Smeets, 1994; Marín et al., 1995, 1997, 1998a, b, c; González et al., 1999, 2014; Smeets and González, 2000; Moreno and González, 2007; Moreno et al., 2017]; Fig. 1c, f). In particular, their detailed work on dopamine cells with ascending projections to the amphibian striatum demonstrated such cells to be located beyond the midbrain tegmentum in all three basal plate divisions of P1 through P3 (VTA/SN in Fig. 1f; [Marín et al., 1995, 1997, 1998a, b]). This was a cornerstone of the new concept of a mesodiencephalic ascending dopamine system also in amniotes (A8-A10 in Fig. 1d, discussed in [Vernier and Wullimann, 2009]; see most recently also [Fougère et al., 2021]). The amphibian work helped to understand our related data in adult zebrafish brain where we showed that dopamine cells in TPp (small round and pear-shaped cells) project to the subpallium [Rink and Wullimann, 2001] and which we interpreted as the diencephalic portion of the mesodiencephalic dopamine system (i.e., amniote A8/9/10; Fig. 1d). Functional support for this comes from the fact that these ascending zebrafish dopaminergic projection neurons have been shown to be glutamatergic, whereas most other zebrafish dopamine cells co-express GABA instead [Filippi et al., 2014]. Most recently, we showed that these zebrafish ascending dopamine cells derive directly from *sonic hedgehog* expressing cells [Wullimann and Umeasalugo, 2020], a feature they share with the entire mesodiencephalic complex in amniotes [Joksimovic et al., 2009; Blaess et al., 2011; Hayes et al., 2011]. However, there is an alternative interpretation of related data in the early zebrafish larva [Tay et al., 2011; Filippi et al., 2014] which proposes that these zebrafish ascending dopaminergic cells correspond to amniote A11 group, mostly based on a shared *Otp* expression (for further discussion of this controversial issue, see my reviews of the dopaminergic ascending system in a comparative phylogenetic context [Wullimann, 2011, 2014]).

Finally, the author returns to the finer genoarchitecture of the basal zebrafish diencephalon by discussing what is known about downstream transcription factor expression. The paired-box bene *Pax7* is an established selective marker gene for periventricular cell masses of bP3 in various sarcopterygians, such as salamanders, frogs, lungfishes, and amniotes [Moreno et al., 2014, 2017]. Recently, *Pax7* was similarly reported in bP3 in larval zebrafish [Schredelseker and Driever, 2020]. Additionally, as in sarcopterygians [Moreno et al., 2014, 2017], zebra­fish *Pax7a* positive cells extend also into the mammillary hypothalamus (see previous section). In contrast, Pax6 is expressed additionally in zebrafish only very laterally in the posterior tuberculum within the early preglomerular complex, and not in the periventricular midline [Wullimann and Rink, 2001; Lauter et al., 2013]. Thus, the *Pax6/Pax7* analysis in Schredelseker and Driever [2020] might potentially be misleading by exclusively using sagittal views which do not allow to evaluate the very different mediolateral positions of the two zebrafish *Pax* genes expression domains as transverse views would.

Lauter et al. [2013] provided a fine prosomeric analysis in the larval zebrafish. Consistent with our proliferation marker studies, as well as bHLH gene expression and phenotypic (TH, *Gad67*, GABA, dopamine; see citations above) analyses, these authors explored downstream of bHLH genes various developmentally relevant transcription factor coding homeobox (*Dbx1a, Dbx2a*, *Emx2*, *Nkx2.1, Nkx2.2, Nkx6.1, Nkx6.2, Six3a*), paired-box (*Pax3a*, *Pax6a, Pax7a*), and LIM-homeobox (*Lhx1a, Lhx5, Lhx9*) genes, plus distal-less homeobox gene *Dlx2a*, forkhead gene *Foxa2*, and signaling factor coding gene *Shha.* They arrived at a genoarchitectonic characterization of three neuromeric partitions each in the alar and basal plate of P1 through P3, and what is more, they provided intraprosomeric genoarchitectonic subdivisions (e.g., three alar pretectal domains, as seen in chicken and mouse [Ferran et al., 2007, 2008, 2009]). Other examples include the exclusive diencephalic expression of *Dlx2a* in the ventral thalamus (prethalamus), but expression of *Pax6* in all three alar diencephalic divisions as we previously described ([Wullimann and Rink, 2001]; Fig. 3e). The additionally investigated bHLH gene *Olig3* is only expressed in the dorsal thalamus. Peculiar in the light of what was discussed above is that *Pax7* expression is only reported in alar pretectum but not in basal P3 [Lauter et al., 2013]. This point needs re-examination in the zebra­fish as the study of *Pax7* might shed light on the derivatives of the large proliferation zone of the teleostean most anterior basal diencephalon (i.e., PTv/bP3).

## Epilogue

In the foregoing, the author explains how the neuromeric/prosomeric model [Puelles and Rubenstein, 1993] has generally been applied to the teleost fish brain. Beyond that, the model has been instrumental for understanding three exemplary topics where teleostean typical features pose particular interpretatory problems. These include the early larval migration areas M1 through M4, the identification of the alar versus basal hypothalamus and hypertrophy of basal hypothalamus, as well as the special situation in the basal diencephalon regarding dopamine cells. In all these cases, a sound comparative neurobiological interpretation was critically supported by applying the neuromeric model. Furthermore, throughout the manuscript, comparative developmental and organizational aspects of the teleostean amygdala are highlighted, in particular the subpallial amygdala.

## Conflict of Interest Statement

The author has no conflicts of interest to declare.

## Funding Sources

The funding sources of my own laboratory's work has been given in the original research papers which are all cited in the appropriate places and need not be repeated in this review. Open access funding is enabled and organized through Projekt DEAL with the Max-Planck-Institute for Biological Intelligence (i.F.).
